# Associations between maternal adiposity and appetite-regulating hormones in human milk are mediated through maternal circulating concentrations and might affect infant outcomes

**DOI:** 10.3389/fnut.2022.1025439

**Published:** 2022-11-04

**Authors:** Sophie Hilario Christensen, Jack Ivor Lewis, Anni Larnkjær, Hanne Frøkiær, Lindsay H. Allen, Christian Mølgaard, Kim F. Michaelsen

**Affiliations:** ^1^Department of Nutrition, Exercise and Sports, Faculty of Science, University of Copenhagen, Frederiksberg, Denmark; ^2^Department of Veterinary and Animal Science, Faculty of Health and Medical Sciences, University of Copenhagen, Frederiksberg, Denmark; ^3^Western Human Nutrition Research Center, Agricultural Research Service (USDA), Davis, CA, United States

**Keywords:** human milk, infant growth, body composition, maternal adiposity, appetite-regulating hormones, leptin, insulin, adiponectin

## Abstract

**Background:**

Appetite-regulating hormones (ARH) in human milk (HM) are suggested to affect infants’ milk intake and possibly infant growth. Maternal adiposity might contribute to higher levels of ARH in HM, either from the mammary gland or from raised circulating levels due to higher adiposity. Counterfactual-based mediation analysis can define indirect and direct effects between HM ARH and maternal and infant factors, and might be an important tool when investigating the mother-milk-infant triad.

**Objective:**

We aim to investigate whether potential associations between (1) maternal adiposity and HM ARH and (2) HM ARH and infant milk intake and growth are mediated through maternal and infant plasma ARH, respectively.

**Materials and methods:**

Maternal and infant anthropometry and body composition, HM and blood samples were collected from 223 mother-infant dyads participating in the Mother, Infant and Lactation Quality study at three postpartum visits from 1 to 8.49 months. Leptin, insulin and adiponectin were analyzed using immunoassays. Mediation analyses using linear mixed-effect models were applied to investigate the direct and indirect effects through maternal and infant plasma hormone concentrations.

**Results:**

A positive association between maternal body-mass-index (BMI) and HM leptin was mediated by maternal plasma leptin by 29% when fixing BMI to < 25 kg/m^2^, and through 51% when fixing BMI to ≥ 25 kg/m^2^ (*p*_interaction_ < 0.01). There was no mediated effect through plasma insulin in the association between BMI and HM insulin (*p* = 0.068). We found negative and positive associations between HM insulin and total milk intake and infant weight, respectively, however, these diminished in mediation analyses with reduced sample sizes.

**Conclusion:**

Our main results suggest that the association between maternal adiposity and HM leptin was mediated through circulating leptin to a stronger degree for mothers with overweight compared to mothers with normal-weight. This indicates that excess maternal adiposity, and the resulting rise of circulating leptin and possible concomitant low-grade inflammation, may be reflected in HM composition.

**Clinical trials registry number:**

NCT03254329.

## Introduction

The World Health Organization (WHO) recommends exclusive breastfeeding (EBF) for six months and continued breastfeeding up to 2 years to ensure optimal nutrition for the newborn infant (1). Current literature additionally posits that breastfeeding has beneficial short- and long-term effects on both mother and infant (2) including a reduced risk of overweight and obesity later in life (3, 4). The hormones leptin, insulin and adiponectin in human milk (HM) are suggested to affect infants growth perhaps through appetite-regulation (5). The adipokines leptin and adiponectin are secreted from the adipose tissue such that circulating levels reflect adiposity in adults as well as in children (6). Gastric leptin increases post-prandially and stimulates hypothalamic receptors in the brain, which further stimulates satiety and thereby promote self-regulation of energy intake (7). Circulating adiponectin has anti-inflammatory properties by promoting insulin sensitivity through decreased energy expenditure and increased glucose uptake (8). Circulating insulin is secreted from the pancreas as a response to postprandial glucose increase and stimulates glucose uptake in the liver and muscle (9).

Furthermore, circulating levels might also reflect obesity-related low-grade inflammation, which is characterized by increased levels of inflammation markers such as C-reactive protein (CRP) and adipokines, such as leptin, secreted from the adipose tissue. Through various mechanisms, low-grade inflammation with elevated levels of such markers contribute to increased risk of type 2 diabetes mellitus (DM), atherosclerosis and non-alcoholic fatty liver disease (10). A study by Savino and colleagues found maternal body mass index (BMI) positively correlated with both maternal serum leptin and HM leptin, which were furthermore correlated with each other (11). Most literature suggests that mammary epithelial cells and possibly adipose tissue secrete the appetite-regulating hormones (ARHs) leptin and adiponectin into the milk (12–14). However, other studies indicate that maternal circulating levels of ARH predict levels in HM (11, 15). The source of HM insulin is mainly hypothesized to be secretion from the epithelial cells of the mammary gland (16) or from maternal plasma (17), and not adipose tissue as such. However, adiposity contributes to insulin resistance increasing plasma insulin (18) and by extension might affect HM insulin. Thus, quantification of the pathways by which ARHs arrive into HM remain to be established.

All three hormones identified in HM have been independently associated with both maternal predictors (5, 19–21) and infant outcomes (11, 22–24). A recently published review concluded an inverse association between HM leptin and infant growth, namely weight and adiposity, based on the 18 included papers (25). Results were mainly seen within the first six months postpartum, where breastfeeding is also most prominent. Inverse associations have similarly been shown between HM adiponectin and infant weight and lean body mass during early breastfeeding (23). Furthermore, Young *et al.* found HM insulin negatively associated with weight-for-length (WLZ) trajectories, but only in infants of normal-weight mothers (26).

Animal studies have shown that labeled milk leptin is absorbed in the gut and can be subsequently detected in the neonatal circulation (27, 28), supporting that intestinal absorption of ARH from HM is a plausible mechanism for the associations seen between HM ARH and infant outcomes. Combined with results showing that maternal adiposity positively associates with HM ARH, these compounds appear to contribute to the complex signaling mechanisms within the mother-milk-infant triad, which covers the factors related to the mother, HM and the infant as well as the associations between these factors (29, 30). Furthermore, breastfeeding studies are limited in their observational nature with confounding making inferences problematic. Most studies rely on traditional approaches when trying to disentangle associations between maternal determinants and HM composition, e.g., adjusting for an intermediate variable. However, this approach may lead to false estimates of the direct and indirect effects. Novel approaches, such as counterfactual-based mediation analysis, identifies the indirect effect through resampling methods. Secondly, a direct effect is derived from the total and indirect effects within the association. This approach has been implemented in a small number of studies investigating associations between maternal predictors of HM composition and infant outcomes (31, 32) and the method improves validity and interpretation of the results (33). We hypothesize that a mediated effect is present through maternal circulation, and the remaining effect can be ascribed as the direct effect covering secretion of ARH from adipose tissue and mammary epithelial cells. Finally, the study exemplifies the use of counterfactual-based mediation analyses in the investigation of the mother-milk-infant triad within HM research.

With this study, we aim to investigate whether potential associations between (1) maternal body composition and HM ARH and (2) HM ARH and infant milk intake and infant growth are mediated through maternal and infant plasma levels of the respective ARHs. To our knowledge, this is the first paper using counterfactual-based mediation analyses to quantify the concentrations of HM ARH mediated through maternal circulation and to investigate mediated effects through infant plasma in the association between HM ARH and infant outcomes.

## Materials and methods

Two-hundred-and-twenty-three Danish mother-infant dyads participating the Mothers, Infants and Lactation Quality (MILQ) study (H-17015174) were included in the present study. The MILQ study is an international multi-center cohort study with the overall aim of developing reference values for micro- and macronutrient concentration in human milk (HM), and is described in detail elsewhere (34).

### Participants

Pregnant women were recruited from hospitals in the Copenhagen area and were included before entering the third trimester of pregnancy (<28th week of gestation) after written informed consent was obtained. Participants were screened according to maternal inclusion and exclusion criteria. Inclusion criteria included being 18–40 years old with a pre-pregnancy BMI of 18.5–29.9 kg/m^2^. They should be non-smokers, have a low intake of alcohol (<5 units per week) and fortified foods (<3 times per week), and should not follow a vegan or macrobiotic diet. Women were excluded if they developed gestational diabetes or preeclampsia during pregnancy, or had anemia, unless they were willing to take iron supplements. Women were informed that they should avoid taking multivitamin supplements and they were offered telephonic breastfeeding counseling by an International Board Certified Lactation Consultant to support WHO’s recommendation of EBF for six months.

Infants were screened telephonically between 2 and 3 weeks after birth. Inclusion criteria for the infants included being born at term (gestational week 37–42) with a birth weight between 2500 and 4200g and without having illnesses affecting growth, development or breastfeeding. Infants were excluded if they were not EBF at Visit 2 (V2) or had ceased breastfeeding completely at Visit 3 (V3). In EBF, formula was allowed only for the first week after birth and sugar water was allowed for analgesic purposes for blood sampling.

### Study design

Participants collected a colostrum sample at birth (Visit 1 = V1) and attended three postpartum visits during the study period; 1.0–3.49 months (V2), 3.5–5.99 months (V3), and 6.0–8.5 months postpartum (Visit 4 = V4). All postpartum visits took place at the Department of Nutrition, Exercise and Sports, University of Copenhagen in Denmark. Visits 2–4 were divided into 3-week windows to which participants were evenly distributed such that data was collected at all ages from 1 to 8.49 months postpartum. Data collected at the three postpartum visits (V2–V4) included height, weight and body composition of mother and infant, maternal and infant blood samples and human milk samples. Daily infant milk intake at each of the three visits was estimated using 24-h test weighing. Information regarding birth, sociodemographic characteristics, maternal and infant morbidity and breastfeeding practices was obtained using questionnaires (34).

### Data collection

#### Maternal and infant anthropometry and body composition

Pre-pregnancy weight and height were self-reported and pre-pregnancy BMI was calculated as weight divided by the squared height (kg/m^2^). Maternal weight and body composition were measured at V2–V4 using a digital scale with an inbuilt bioelectrical impedance function (Tanita-MC780MA; Tanita Corporation, Tokyo, Japan). The measurement was performed with an empty bladder with only light clothes worn, of which the weight was estimated and corrected for. Fat mass (FM) was estimated using the internal Tanita algorithm (not publically available) incorporating sex, age and height. Maternal height was measured at V2 to the nearest millimeter (mm) using an average of three measurements. FM and fat free mass (FFM) indexes (FMI and FFMI, respectively) were calculated by dividing FM and FFM (in kg) by the participant’s squared height (in m).

Infant birth weight and length were self-reported from hospital records, and infant weight, length and body composition were measured at V2–V4. Infant weight was measured using a digital scale (Tanita-BD815MA; Tanita Corporation, Tokyo, Japan) and length was measured to the nearest mm with a portable measuring board (SECA 416; SECA, Hamburg, Germany) using an average of three measurements. Infant weight and length were measured without clothes or diaper. Infant body composition was estimated using bioelectrical impedance (Bodystat-500; Bodystat Ltd., Isle of Man). Two electrodes were placed on the right hand and two on the right ankle, and an average of three measurements was used. Age-appropriate equations generated by Lingwood *et al.* were used to convert whole-body impedance to FFM, which was subtracted from infant weight to give FM (35). Indexes of FM and FFM were calculated as mentioned above.

#### Maternal and infant blood sample collection

For the purpose of the entire MILQ study, non-fasting blood samples were collected from both mother (18 mL) and infant (6 mL) at V2–V4 and 200 μL was used for analysis of this present study. All infants had blood samples taken at V2, however, to reduce discomfort for the infants, only 50% had blood samples taken at V3 and the other 50% had blood samples taken at V4. Whole blood was collected by venipuncture into a vacutainer collection tube (K_2_EDTA 3 mL for infants and K_2_EDTA plasma mineral trace free for mothers), from which plasma was obtained after centrifugation (1500xg for 10 min. 4°C). Samples were stored in 2 mL amber screw cap tubes at –70 to –80°C until analysis. Local analgesics was offered for the infants prior to sample collection.

#### Human milk collection

Mothers collected a colostrum sample (1 mL) in a small, clean tube provided by study staff. The sample was collected by hand expression 24–72 h after birth, either at home or at the hospital. The sample was protected from sunlight using tinfoil and stored in the home freezer (–18°C) or at the hospital for a maximum of 3.49 months before being transferred to the university freezer (–80°C) until analysis.

Human milk was collected as full breast expressions using an electric breast pump and 250 mL collection bottles (Medela Symphony; Medela; Baar, Switzerland). Samples were collected around 09:00 or around 14:00 h from the breast not last used for feeding. The breast was cleaned with an alcohol-free wet wipe prior to sample collection and a sample of 30 mL was retained from the full breast expression in an amber 50 mL polypropylene tube. The whole milk sample was mixed and homogenized and aliquoted in 2 mL amber screw cap tubes immediately hereafter and stored in the freezer (–80°C) until analysis.

#### Analysis of human milk and plasma samples

Leptin and insulin in both whole milk and plasma were measured using MSD U-plex multiplex immunoassays (Meso Scale Diagnostics, Rockville, USA). Adiponectin in both whole milk and plasma was measured by sandwich enzyme-linked immunosorbent assay (sELISA) using a human adiponectin duoset (DY1065) from R&D (Biotechne, Minneapolis, MN, USA). Assays were performed according to manufacturer protocols. Plasma insulin and leptin were added undiluted to the wells while the respective HM samples were diluted 1:2 in in the provided diluent. For analysis of adiponectin, HM was diluted 1:10 and plasma samples 1:20,000 in PBS with 1% BSA, pH 7.2–7.4. A minor number of samples were out of the range and were re-analyzed at a higher or lower dilution.

Detection limits of insulin, leptin and adiponectin were 12, 11 pg/mL and 5 ng/mL, respectively. For non-detectable (NA) data, half of the lower cut-off concentration of the specific hormone was used for statistical analyses. Raw data prior to data imputation can be seen in Supplementary Table 1 including number of NA samples. An internal reference sample was prepared by pooling aliquots of 80 plasma samples and included in duplicates on each plate. The obtained values were used to determine assay variability. The intra assay coefficient of variability (CV) for insulin, leptin and adiponectin was 8.5, 9.5, and 11%, and inter assay CV-values were 18, 20, and 29%, respectively.

Energy in HM was calculated using macronutrients concentrations. Whole milk samples (500 μL) were analyzed using near infrared spectroscopy by measuring absorbance for fat, protein, and carbohydrates as per the manufacturer’s protocol (SpectraStar XT; Unity Scientific, Milford, MA, USA). Energy concentration was estimated using the macronutrients energy content per gram (4 calories/g for carbohydrate and protein; 9 calories/g for fat). The machine was originally calibrated with known human milk samples from a reference laboratory and subsequently tested with an in-house milk pool with every set of analysis. Samples were heated to 35°C and homogenized in dim light before analysis. The sonicator tip and measuring cup was cleaned firstly with deionized water and secondly with methanol in between scans, according to manufacturer’s protocol.

#### Infant milk intake

Human milk intake was measured by 24-h test weighing using a digital scale (ADE M101000-01; ADE GmbH & Co., Hamburg, Germany) with an accuracy of 5 g for weights below 10 kg and 10 g for weights above 10 kg. Participants received the scale after attending a visit and completed a weighing protocol within 7 days after the visit. The weighing took place from 08:00 to 07:59, plus one extra weighing to define the 24 h. The mother was informed to weigh the infant with the same clothes and diaper at the two weighings in order to get a correct estimate of milk intake defined by the weight difference between the two feeds. The mother logged the time and infant weight before and after the feed and which breast was used. Logs were double-entered electronically by study staff. Single feeds > 400g were regarded as being unrealistically high, and marked as missing. Logs with > 3 missing feedings were regarded insufficient and were discarded. For participants with ≤3 missing feedings, missing data was imputed using hot deck imputations based on neighboring weights from the same participant.

### Statistical analysis

Continuous characteristics are given as mean ± standard deviation (SD) when normally distributed and as median ± inter quartile range (IQR) when non-normally distributed. Categorical characteristics are given as counts and percentages unless otherwise stated. Interactions, collinearity and assumptions of linearity, normality and equal variance of residuals were checked before reporting the final models. Model estimates of log-transformed variables were back-transformed and expressed as percent difference.

Concentrations of ARH in HM as well as maternal and infant plasma across lactation were described using linear mixed-effect models applying a robust unstructured covariance structure. Models were adjusted for infant sex and a centered age derived by subtracting infant age at a visit from the mean age of all infants at that visit.

**FIGURE 1:**
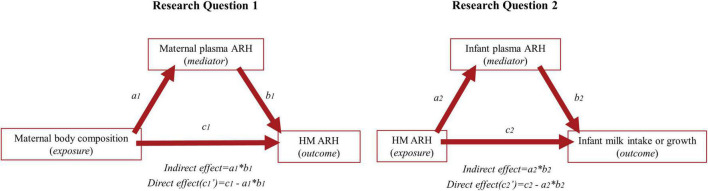
Conceptual diagram of the investigated mediation analyses of Research Question (RQ) 1 **(left)** and 2 **(right)**. Maternal body composition (exposure) in RQ1 includes body mass index and fat mass index. Outcome in RQ1 and exposure in RQ2, HM ARH, includes human milk concentrations of leptin, insulin and adiponectin. Outcome in RQ2 includes infant milk intake (total 24-h milk intake and milk intake per feed), and infant growth (infant weight, length, fat mass index and body fat percentage). a_1_ describes the estimate for the influence of exposure on mediator, b_1_ indicates the estimate for the influence of the mediator on the outcome, and c_1_ describes the total effect (direct + indirect) of exposure on outcome. The indirect effect (a_1_b_1_) can be estimated by multiplying a_1_ and b_1_ and the direct effect can be estimated by c_1’_ = c_1_ - a_1_b_1_.

Counterfactual-based mediation analyses using linear mixed-effect models were applied to investigate the following two research questions (RQ); (1) whether potential associations between maternal body composition (BMI and FMI) and HM ARH (leptin, insulin and adiponectin) are mediated through maternal plasma markers of the respective hormones, and (2) whether potential associations between the HM ARHs and either infant milk intake (total 24-h intake or intake per feed) and/or infant growth (weight, length, FMI and body fat %), respectively, are mediated through infant plasma levels of the respective hormones (Figure 1). Exposure-mediator interactions were tested in the initial models. If significant, the exposure was converted into a categorical variable, i.e., BMI < 25 or ≥ 25 kg/m^2^ and FMI ≤9 or > 9 corresponding to BMI groups (36), and the interaction term was included in the mediation analyses. Definitions of total, direct and indirect effects for categorical exposure variables can be found in Supplementary Table 2. Fixed effects further included infant sex, visit and mean-centered age for RQ1. For RQ2, separate mediation analyses investigated either milk intake as outcome, which included HM energy concentration, infant sex, weight and age as additional fixed effects, or infant growth as outcome, including HM energy concentration, total milk intake, birth weight, infant sex and age as additional fixed effects. Subjects were included as random effects in all models. Possible confounding variables mentioned as fixed effects were chosen *a priori* based on current literature and plausible biological associations.

The following steps comprised the mediation analysis: (1) testing if the exposure was significantly associated with the outcome (total effect = c_1_), (2) testing if the exposure was significantly associated with the mediating variable (a_1_) in a “*mediator model*,” (3) testing if the mediating variable was significantly associated with the outcome (b_1_) in an “*outcome model*,” and (4) testing if the mediated (indirect) effect (a_1_b_1_) was significant. The indirect effect can also be defined as the product of a_1_ and b_1_ and the total effect is equal to the sum of the direct and indirect effect (Figure 1). The significance of the indirect effect was tested using The Monte Carlo Method for Assessing Mediation (37) where a 95% confidence interval (CI) was estimated by resampling of the estimates and distributions of a_1_ and b_1_. Sample size in the initial model, testing the total effect (c_1_), was restricted to include complete cases of included covariates of this model only, whereas sample size in the mediation analyses was restricted to include complete cases of included covariates in all steps of the analyses.

All statistical analyses were conducted using R software (version 4.1.3; R Foundation for Statistical Computing) (38). The *LMMstar* package (39) was used to define linear mixed-effect models with robust unstructured covariance structures to compare HM ARH in colostrum and mature milk. The *lme4* and *mediation* packages (40, 41) were used to define linear mixed-effect models to be used in mediation analyses, and to conduct the mediation analyses, respectively. Sex differences in infant characteristics were tested using a parametric *t*-test for normally distributed data and a Mann-Whitney *U*-test for non-normally distributed data. A (two-sided) *P*-value < 0.05 was considered statistically significant.

## Results

**FIGURE 2:**
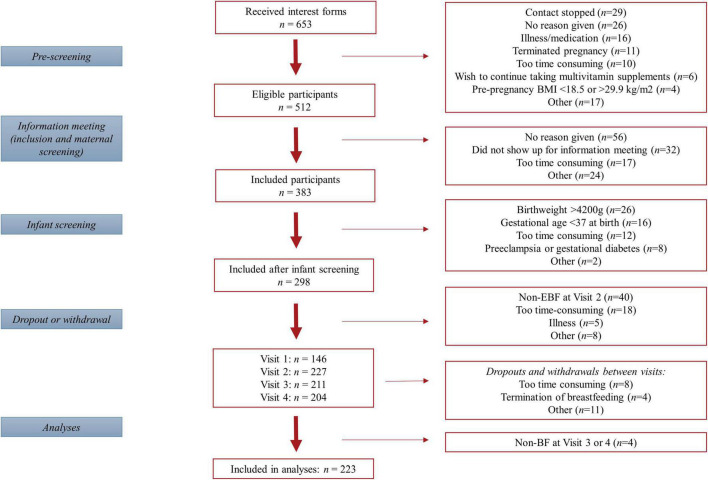
Flow diagram of participants included in the study.

Three-hundred-and-eighty-three women gave informed consent to participation of which *n* = 146 collected a colostrum sample and *n* = 227, *n* = 211 and *n* = 204 completed V2, V3, and V4, respectively. The majority dropped out or were excluded either (a) at birth (*n* = 72) due to exclusion criteria regarding birth weight or gestational age at birth, or (b) before V2 (*n* = 71) because infants were not EBF or parents considered participation too time consuming. For the present analyses, mother-infant dyads were further excluded if they did not breastfeed at any time point (*n* = 4) resulting in *n* = 223 being included in the analyses of which *n* = 204 completed all three postpartum visits (Figure 2). Excluded participants did not differ significantly from the completers with regards to relevant outcomes and/or baseline data (data not shown).

Mothers were 31.5 ± 3.4 years old with a pre-pregnancy BMI of 22.5 ± 2.5 kg/m^2^ and 14% being overweight (BMI ≥ 25 kg/m^2^) before pregnancy. Two thirds had a long education and more than 20% had a household income more than 1 million DKK/year corresponding to about 135,000 euro/year (Table 1). Mothers gained 13.4 ± 4.4 kg during pregnancy and had lost their pregnancy weight gain by the end of the study. Maternal BMI decreased slightly from V2 to V4 (Table 2A) and 31, 5, and 20% were overweight, obese and had excessive fat mass, respectively, at any visit.

**TABLE 1:** Participant characteristics at inclusion.

Maternal characteristics			All (*n* = 223)
Pre-pregnancy BMI (kg/m^2^)			22.5 (2.5)
BMI ≥ 25 kg/m^2^			32 (14)
Gestational weight gain (kg)			13.4 (4.4)
Age (years)			31.5 (3.4)
**Higher education**			
Short (<3 years)			15 (7)
Medium (3–4 years)			51 (22)
Long (>4 years)			161 (71)
Household income (DKK**)			
<500,000			34 (15)
500,000–799,999			74 (33)
800,000–999,999			71 (31)
>1,000,000			48 (21)

**Infant characteristics**	**Males (*n* = 101)**	**Females (*n* = 122)**	**All (*n* = 223)**

Gestational age at birth (weeks)	40.3 (1.1)	40.3 (1.1)	40.3 (1.1)
Birth weight (g)	3,600 (360)	3,440 (353)*	3,510(364)
Birth length (cm)	52.2 (1.9)	51.4 (1.7)*	51.8 (1.9)

Results are given as mean (SD) and *n* (%) for categorical variables.

*Indicates significant difference between sexes.

**7.5 DKK–1.00 euro.

**TABLE 2A:** Maternal anthropometry, body composition and plasma and human milk hormone concentrations at the three postpartum visits.

	V1 (*n* = 146)	V2 (*n* = 223)	V3 (*n* = 210)	V4 (*n* = 202)
Postpartum age (months)	–	2.1 (0.7)	4.6 (0.8)	7.1 (0.7)
Maternal weight (kg)	–	67.3 (8.6)	65.7 (8.8)	64.8 (8.9)
Maternal BMI (kg/m^2^)	–	23.6 (3.0)	23.1 (3.1)	22.7 (3.1)
*BMI* ≥ *25kg/m^2^*	–	61 (28)	52 (25)	43 (22)
Maternal fat mass index	–	6.9 (2.1)	6.5 (2.2)	6.2 (2.2)
*Fat mass index* > *9*	–	37 (17)	30 (15)	20 (10)
Plasma leptin (ng/mL)	–	4.7 [2.1, 12.4]	4.3 [1.6, 10.0]*	5.1 [2.5, 11.1]
Plasma insulin (pg/mL)	–	131 [93, 179]	163 [109, 248]	127.0 [88, 201]
Plasma adiponectin (μg/ml)	–	4.4 [3.1, 5.8]	7.0 [5.0, 9.3]*	4.0 [2.7, 6.8]
Human milk leptin (pg/mL)	419 [192, 807]	90 [10, 252]*	48 [10, 144]*	33 [10, 144]*
Human milk insulin (pg/mL)	416 [263, 659]	202 [153, 265]*	218 [164, 283]	211 [169, 296]
Human milk adiponectin (ng/mL)	8.2 [4.7, 25.8]	2.4 [1.6, 3.7]*	1.9 [1.2, 2.8]	2.0 [1.3, 3.1]
Human milk energy (kcal/L)	–	633 (102)	615 (110)	615 (143)

Results are given as mean (SD) or *n* (%) for counts or as median [interquartile range].

Visit 1 (V1) = 24–72 h, V2 = 1–3.49 months, V3 = 3.5–5.99 months, V4 = 6.0–8.5 months postpartum. Imputations have been made for not assessable values of plasma and human milk leptin, insulin and adiponectin.

*Indicates significant difference from the previous visit.

Infants were born at 40.3 ± 1.1 weeks of gestation with a mean birth weight of 3510 ± 364 g and birth length of 51.8 ± 1.9 cm (Table 1). Infants were exclusively breastfed for 4.6 ± 0.9 months and 94 and 53% were exclusively breastfed at V2 and V3, respectively. At V2, 6% had received minimal amounts (≤1 tablespoon; ≤5times) of water or sugar water. Infants had a mean age of 2.1 ± 0.7, 4.6 ± 0.7 and 7.1 ± 0.7 months when attending V2, V3, and V4, respectively.

We had complete milk volume records for *n* = 204, *n* = 172 and *n* = 148 at V2, V3, and V4, respectively, following imputations as described. Total milk intake was significantly higher for males at V2 only (β = 52.2 mL, *p* = 0.020), whereas milk intake per kg bodyweight was higher for females at V3 (β = 11.6 mL, *p* = 0.0035; Table 2B).

**TABLE 2B:** Infant anthropometry, body composition, milk intake and plasma hormone concentrations at the three postpartum visits.

	V2		V3		V4	
	Males	Females	Males	Females	Males	Females
Age (months)	2.1 (0.7)	2.0 (0.7)	4.7 (0.8)	4.5 (0.7)	7.2 (0.7)	7.1 (0.7)
Weight (g)	5820 (856)	5210 (759)*	7570 (736)	6880 (963)*	8730 (787)	8110 (1060)*
Length (cm)	60.1 (3.0)	58.2 (2.7)*	67.0 (2.5)	64.7 (2.6)*	70.8 (2.4)	68.9 (2.5)*
Fat mass index	3.5 (0.7)	3.5 (0.7)	4.1 (0.9)	4.4 (1.1)*	4.8 (0.9)	5.3 (1.1)*
Fat-free mass index	12.5 (0.7)	11.8 (0.7)*	12.8 (0.7)	11.9 (0.8)*	12.6 (0.8)	11.8 (0.8)*
Body mass index (kg/m2)	16.0 (1.3)	15.4 (1.3)*	16.8 (1.2)	16.4 (1.5)*	17.4 (1.3)	17.1 (1.6)
Body fat %	21.7 (2.9)	22.8 (3.2)*	24.1 (4.3)	27.0 (4.4)*	27.3 (3.2)	30.7 (4.0)*
Total milk intake, 24h (mL)	813 (163)	761 (150)*	844 (191)	840 (176)	641 (203)	600 (239)
Milk intake per feed (mL)	85.5 (22.3)	80.1 (21.3)	91.4 (29.2)	90.2 (23.5)	93.4 (32.2)	87.9 (34.6)
Milk intake (24 h) per kg body weight (mL/kg)	143 (32)	147 (29)	112 (25)	123 (25)*	73 (23)	75 (29)
Plasma leptin (ng/mL)	2.3 [1.5, 3.7]	3.2 [1.6, 8.1]	1.8 [0.9, 3.0]	1.6 [1.3, 4.8]	1.5 [0.4, 3.9]	3.0 [1.6, 5.0]
Plasma insulin (pg/mL)	144 [96, 180]	127 [90, 183]	72 [58, 112]	87 [69, 106]	77 [57, 84]	133 [84, 170]*
Plasma adiponectin (μg/mL)	23.9 [18.2, 30.1]	23.4 [17.3, 27.6]	18.1 [15.2, 21.9]	20.1 [16.2, 22.9]	11.1 [10.0, 13.8]	9.2 [8.8, 12.5]

Results are given as mean (standard deviation) or as median [interquartile range].

Visit 2 (V2) = 1–3.49 months, V3 = 3.5–5.99 months, V4 = 6.0–8.5 months postpartum.

*Indicates significant difference between sexes at each visit.

### Appetite-regulating hormone concentration across lactation

Colostrum samples were collected 2.1 days ± 0.07 postpartum, and median and IQR for leptin, insulin and adiponectin concentrations were 419 pg/mL [192, 807], 416 pg/mL [263, 659] and 8.2 ng/mL [4.7, 25.8], respectively (Table 2A). Concentrations of all three ARH decreased significantly from V1 to V2 (colostrum to mature milk) by 78, 54, and 80% (β = -1.54; β = -0.78; β = -1.64, *p*_all_ < 0.01) and with no difference by infant sex (*p*_interactions_ > 0.05) (data not shown).

In mature milk, median and IQR for leptin concentrations were 90 pg/mL [10, 252] at V2, which halved at each subsequent visit (β_V2–V3_ = β0.71; β_V3–V4_ = -0.69, *p* = 0.04). Insulin concentration was 202 pg/mL [153, 265] at V2 and remained constant to V4 (β = 0.13, *p* = 0.41). Adiponectin concentration was 2.4 ng/mL [1.6, 3.7] at V2 and decreased by 27% from V2 to V3 only (β = -0.33, *p* = 0.02; Table 2A).

### Maternal body composition and appetite-regulating hormones in human milk (RQ1)

**FIGURE 3:**
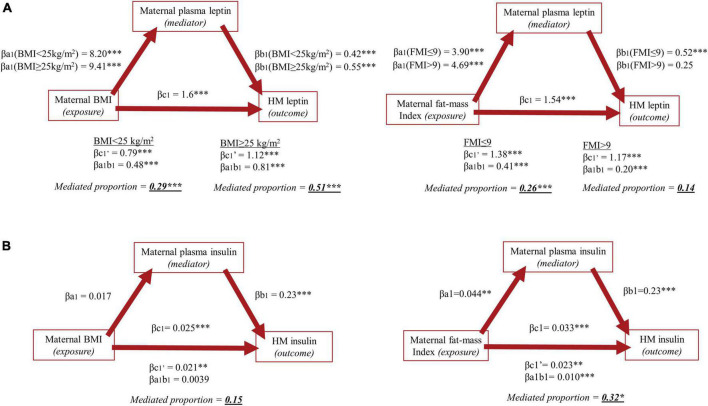
Mediation analyses of Research Question 1 investigating the association between maternal body mass index (left) and fat-mass index (right) as exposure variables and human milk concentrations of **(A)** leptin and **(B)** insulin as outcome variables, with maternal plasma concentrations of the respective hormone as mediating variables. In mediation analyses including HM leptin, an exposure-mediator interaction resulted in categorization of maternal BMI into normal- or overweight (BMI < 25 or ≥ 25kg/m^2^) and FMI into having normal or excessive fat mass (FMI ≤ 9 or > 9) and estimates are given for each group. No exposure-mediator interaction was present in mediation analyses including HM insulin, and the exposure variables were therefore not categorized and estimates are given accordingly.

We found a significant exposure-mediator interaction between maternal BMI and maternal plasma leptin in the initial model associating maternal BMI with log-HM leptin (Table 3). Specifically, the strength of the positive association between maternal BMI and log-HM leptin increased with higher plasma leptin. Consequently, maternal BMI was categorized into mothers with normal-weight (BMI < 25kg/m^2^) or with overweight (BMI ≥ 25kg/m^2^) and the interaction term (BMI_categorical_*plasma leptin_log_) was included in the mediation analyses as described. As such, we found 400% higher leptin in milk of mothers with compared to without overweight (β_c1_ = 1.6, *p* < 0.01). The effect was mediated through maternal plasma leptin by 29% when BMI was fixed to normal-weight (βa_1_b_1_ = 0.48; *p* < 0.01) and by 51% when BMI was fixed to overweight (βa_1_b_1_ = 0.81; *p* < 0.01; Figure 3 and Supplementary Table 3). Furthermore, we found an interaction between maternal BMI and infant sex in the initial association between maternal BMI and log-HM leptin. This resulted in mothers with female offspring had a 30% higher HM leptin concentrations compared to mothers of males in the initial model (β = 0.28; *p* = 0.057; Table 3). An exposure-mediator interaction in the association between maternal FMI and log-HM leptin (β = 0.080; *p* = 0.002) (data not shown) resulted in a categorization of mothers into having normal (FMI ≤ 9) or excessive (FMI > 9) fat mass as described. The association was only mediated through maternal plasma leptin when FMI was fixed to normal fat mass by 26% (βa_1_b_1_ = 0.41; *p* = 0.002), but not when fixed to excessive fat mass (βa_1_b_1_ = 0.20; *p* = 0.19; Figure 3 and Supplementary Table 3). We did not find an interaction between maternal FMI and infant sex in the initial association between maternal FMI and log-HM leptin.

**TABLE 3:** Model estimates of the initial testing of the total effect (c_1_) of maternal BMI on human milk leptin, insulin and adiponectin.

	Log-HM leptin (*n* = 221)		Log-HM insulin (*n* = 221)		Log-HM adiponectin (*n* = 219)	
	Estimate	95% CI	Estimate	95% CI	Estimate	95% CI
***(a) Maternal BMI***						
Maternal BMI (kg/m^2^)	−0.37*	[–0.71 to (–0.02)]	–	–	–0.00	[–0.03 to 0.02]
*Maternal BMI (kg/m^2^) for mothers of males* ^α^	–	–	0.0079	[–0.01 to 0.03]	–	–
*Maternal BMI (kg/m^2^) for mothers of females* ^α^	–	–	0.038***	[0.02 to 0.06]	–	–
Infant sex (females vs. males)	0.28*	[0.00 to 0.5]	−0.71*	[–1.36 to (–0.06)]	–0.04	[–0.18 to 0.10]
Log-plasma leptin	−0.95*	[–1.80 to (–0.10)]	–	–	–	–
Maternal BMI*log-plasma^β^	0.060**	[0.02 to 0.10]		–	–	–

^α^Estimates of the influence of maternal BMI on HM insulin are shown separately for each infant sex instead of the interaction term between maternal BMI and infant sex.

^β^Exposure-mediator interaction term between maternal BMI and maternal plasma concentration of each hormone.

All models were adjusted for visit and mean-centered age.

BMI, body mass index; CI, confidence interval; HM, human milk.

*p* < ***0.001; *p* < **0.01; *p* < *0.05.

There was no exposure-mediator interaction in the association between maternal BMI and neither log-HM insulin nor log-HM adiponectin (*p*_both_ > 0.05; Figure 3 and Supplementary Table 3). The association between maternal BMI and log-HM insulin was mainly influenced by a direct effect of BMI of 84% (β_c1’_ = 0.021; *p* < 0.01) and with an indirect effect of 15% (β_a1b1_ = 0.0039; *p* = 0.068). Thirty-two percent of the association between maternal FMI and log-HM insulin (β_c1_ = 0.033, *p* < 0.01) was mediated through maternal plasma insulin (β_a1b1_ = 0.010, *p* < 0.01). Lastly, we found a significant interaction between maternal BMI and infant sex in the initial model including insulin (β = 0.030; *p* = 0.03; Table 4), showing a significant increase in HM insulin of 4% per unit increase in BMI for mothers of female offspring (β = 0.04; *p* = 0.0001), but not for mothers of male (β = 0.008; *p* = 0.5).

**TABLE 4:** Initial testing of the total effect (c_1_) of leptin, insulin and adiponectin concentrations on (a) total milk intake, (b) milk intake per feed, (c) infant weight, (d) length, (e) fat mass index and (f) body fat percentage, respectively.

	Log-HM leptin			Log-HM insulin			Log-HM adiponectin		
	*n*	Estimate	95% CI	*n*	Estimate	95% CI	*n*	Estimate	95% CI
(a) Total milk intake (mL) *for males*^α^	196	–5.1	[–14.6 to 4.4]	196	−89.8*	[–152.2 to (–27.4)]	197	–20.5	[–45.5 to 4.5]
(a) Total milk intake (mL) *for females*^α^	–	–	–	196	21.2	[–32.0 to 74.3]	–	–	–
(b) Milk intake per feed (mL)	196	1.1	[–0.6 to 2.8]	196	4.0	[–3.4 to 11.5]	197	–0.6	[–4.2 to 3.0]
(c) Infant weight (kg)	196	2.7	[–25.1 to 30.4]	196	140.2*	[23.3 to 257.2]	197	–37.4	[–111.7 to 36.9]
(d) Infant length (cm)	196	0.06	[–0.02 to 0.2]	196	0.2	[–0.2 to 0.6]	197	–0.18	[–0.4 to 0.06]
(e) Infant FMI	161	–0.0	[–0.05 to 0.04]	161	0.07	[–0.1 to 0.3]	162	–0.05	[–0.2 to 0.06]
(f) Infant body fat %	161	0.01	[–0.2 to 0.2]	161	0.03	[–0.6 to 1.1]	162	–0.17	[—0.7 to 0.3]

Log-HM hormones describes the exposure and (a–f) describes the outcomes in the models. Models (a, b) are adjusted for HM energy concentration, infant sex, weight and age, and models (c, e) are adjusted for HM energy concentration, infant sex, birth weight, and total milk intake.

^α^There was an interaction between log-HM insulin and infant sex, and the estimate of log-HM insulin is indicated separately for each sex for HM insulin only, whereas estimates of log-HM leptin and log-HM adiponectin is presented for both sexes in (a).

CI, confidence interval; FMI, fat mass index; HM, human milk.

*p* < ***0.001; *p* < **0.01; *p* < *0.05.

Human milk adiponectin was not influenced by either maternal BMI (β = -0.0; *p* = 0.4; Table 3) or FMI (β = 0.0, *p* = 0.7) (data not shown).

### Appetite-regulating hormones in human milk and infant outcomes (RQ2)

We found a negative association between log-HM insulin and total milk intake in male offspring (β = -89.8, *p* = 0.005) resulting in 8.5 mL lower milk intake per 10% increase in HM insulin, but not in female (β = 21.2, *p* = 0.43). Furthermore, a positive association was seen between log-HM insulin and infant weight (β = 140.2, *p* = 0.02; Table 4) resulting in a 13g increase in weight per 10% increase in HM insulin. However, the associations disappeared in mediation analyses when sample size was reduced from *n* = 197 to *n* = 123–135 due to restrictions to number of complete cases in the mediation analyses (data not shown).

There was no association between log-HM leptin and either total milk intake or milk intake per feed (*p*_both_ > 0.05). Infant weight and mean-centered age, i.e., being older at the visit, were associated with either total milk intake or milk intake per feed for all three ARH (*p*_all_ < 0.05) (data not shown). No associations were found between either log-HM leptin or adiponectin and either of the infant growth outcomes, respectively (*p*_all_ > 0.05; Table 4).

## Discussion

We found concentrations of all three ARH decreasing from colostrum to mature milk, which is in accordance with previous literature (6). A large gap between tight junctions in the mammary epithelial cells due to inflammation when the first milk “comes in” creates space for larger compounds to enter the milk (12, 42). Furthermore, we found leptin in mature milk decreasing throughout lactation, whereas adiponectin decreased until 3.5–6 months, and remained constant to 6–8.5 months, as did insulin in mature milk throughout lactation.

A study by Bronsky *et al.* found median HM leptin falling from 0.30 to 0.05 ng/mL from 0 to 12 months (43), which support our findings of declining leptin concentrations across lactation. This is further supported by a study by Dadres and colleagues reporting declining HM leptin concentrations from 1 to 3 months (19). However, two separate studies find HM leptin nearly constant (44, 45). The study by Dadres and colleagues furthermore found HM adiponectin declining from 1 to 3 months, but constant levels of HM insulin from 1 to 3 months, which is in accordance with our findings. In the present study, neither time of sample collection nor time since last feed affected any of the HM ARH concentrations.

### Maternal body composition and appetite-regulating hormones in human milk (RQ1)

We found maternal BMI positively associated with both HM leptin and insulin, but not with HM adiponectin. The association with HM leptin was mediated through plasma leptin concentrations to a stronger degree when BMI was set to ≥ 25kg/m^2^ compared to < 25kg/m^2^. There was no mediated effect through plasma insulin in the association between maternal BMI and HM insulin, but a mediated effect was present when using FMI as exposure.

Studies have suggested that leptin is secreted from secretory cells in the membrane of the mammary glands (46), from the adipose tissue or that it traverses the basolateral membrane of the epithelial cells from the blood stream to the milk (47). Our results suggest that the route through maternal circulation is more prominent in mothers with overweight compared to mothers with normal-weight. The percentage at which the association between maternal BMI and HM leptin can be explained by the circulating levels is about 50% for mothers with overweight and about 30% for mothers with normal-weight. Accordingly, a direct effect is more prominent in mothers with normal-weight compared to those overweight. The estimated direct effect of maternal adiposity covers other mechanisms than through maternal circulation, which to our knowledge include secretion from the mammary gland and the adipose tissue. However, the latter two mechanisms are not distinguished in the present analysis. A greater mediated effect seen in mothers with overweight support the influence of adiposity on low-grade inflammation in these mothers, in whom enlarged adipocytes are suggested to produce more leptin. The adipocytes are known to further secrete inflammatory cytokines such as tumor-necrosis factor-α (TNF-α) and interleukins, which upregulate mRNA expression of leptin in the adipocytes, creating a loop of compounding signaling components maintaining a chronic inflammatory state (10, 48). However, inflammation markers such as TNF-α and CRP were not measured in the present study, which could be relevant to include for assessment of the inflammatory condition and justifying the use of leptin as a marker of low-grade inflammation. In mediation analyses associating FMI with HM leptin, a mediated effect was only present when FMI was fixed to ≤9 compared to >9. More mothers categorized as overweight (≤28%) than categorized as having excessive fat mass (≤17%) reduces power and could explain the absence of a mediated effect.

Lastly, the direct and total effects of maternal BMI and FMI were considerable higher for leptin compared to insulin, which shows a distinctly stronger influence of maternal adiposity on HM leptin compared to HM insulin. This can possibly be explained by leptin secretion directly from the adipose tissue, possibly also locally in the breast.

The origin of HM insulin is less investigated, but a study by Whitmore *et al.* suggests that HM insulin is transported from the circulation as they found insulin in milk of mothers with type 1 DM originating from exogenous insulin administration (17). On the contrary, HM insulin is also suggested to originate from the epithelial cells of the mammary gland (16). As we found no prominent indirect effect through maternal plasma insulin, our results support the latter findings. In addition, insulin is the only hormone of the three investigated, which we found in higher concentrations in HM compared to plasma. This has been shown previously (49) and supports that insulin is secreted into the milk through other mechanisms than through circulating insulin, possibly from the epithelial cells. Our results showed a mediated effect through plasma insulin when using FMI as the exposure. Although the adipose tissue does not secrete insulin, adiposity contributes to insulin resistance and might explain the mediated effect through plasma. All biological samples were non-fasting due to ethical concerns for lactating mothers, however, adjusting for time since last feed did not change the results.

We found no association between maternal body composition and HM adiponectin. Aside from one study which reported positive associations between maternal body composition and HM adiponectin (50), most recent studies found positive correlations with maternal circulating adiponectin and not body composition (23). This could indicate that HM adiponectin to some degree originates from the circulation, however, this was not investigated in the present study.

Leptin in HM of mothers with female compared to male offspring was 30% higher in the initial model, however, only borderline significant. The results support the findings by Weyermann and colleagues, who reported lower HM leptin levels from women with male compared to female offspring (51). Two separate studies found higher circulating leptin concentrations in breastfed females compared to breastfed males (52, 53), which could be explained by increased ingested leptin. Whether higher circulating leptin for females is caused by increased ingestion from HM, or if the milk leptin is “tailored” to the sex of the infant is not yet established. Other possible explanations could be either a larger fat mass for females compared to males (54) or by testosterone in males suppressing leptin secretion (55). A study by Fields *et al.* found higher HM insulin in mothers with compared to without obesity, but only in mothers with female offspring (56). A similar interaction between BMI and infant sex for HM insulin was also found in the present study. Fields and colleagues suggest that hormone secretion by the placenta during pregnancy differing by infant sex might contribute to these results. Infant-driven factors could similarly affect the association, e.g., lower milk intake for female offspring increasing insulin concentration in the milk if total insulin was constant across individuals. In the present study, mothers with female compared to male offspring had a 4% greater increase in HM insulin per increase in BMI unit. Furthermore, HM insulin across individuals was constant in mature milk, so a lower milk intake for female offspring could have affected the association. Estimates in the study by Fields *et al.* were considerably larger with a 179% higher HM insulin in mothers with obesity with female offspring compared to male offspring. Compared to our results, this might indicate that the interaction with infant sex has a greater effect the higher the BMI.

### Appetite-regulating hormones in human milk and infant outcomes (RQ2)

Insulin concentrations in HM was negatively associated with total milk intake, but only in male offspring, and not female, suggesting that HM insulin influence appetite-regulation in male and female offspring differently. Insulin in HM was furthermore positively associated with infant weight with 13g increase in body weight per 10% increase in HM insulin. The study by Young *et al.* report inverse associations between HM insulin and WLZ *z*-scores in infants of mothers with normal-weight (26), which indicate that insulin either affected infant length positively or infant weight negatively. The latter being opposite to our findings and we did not find positive associations with length. However, lack of associations seen in infants of mothers with obesity might be explained by a greater infant weight, which could mask the association. A study by Chan *et al.* found that intermediate HM insulin concentrations were associated with lower WLZ at four months (21) in a U-shaped association, but Fields *et al.* found no associations between HM insulin and infant body composition during the first six months after birth (56). These studies all have sample sizes ≤52 and results need to be confirmed in other studies. However, both associations with HM insulin disappeared in mediation analyses, where sample size was reduced.

We found no associations between HM leptin concentrations and either total milk intake or milk intake per feed, but as expected, infant weight and mean-centered age were independently associated with the two intake variables. However, it is likely that milk intake is the driver of infant weight and not the other way around. We did not find any associations between neither HM leptin nor adiponectin and any of the growth outcomes, and the literature states conflicting results within this research area (25). Galante and colleagues found leptin concentrations in HM 5 days postpartum positively associated with infant fat mass at discharge for moderate-late preterm infants, however HM leptin was negatively associated with fat mass at 4 months’ corrected age (57). A considerable unknown factor is the plausible mechanism assumed when posing hypotheses regarding HM composition and associations with infant outcomes. Although gastrointestinal uptake of ingested leptin and adiponectin has been shown in animal studies (14, 27, 28), the mechanism has not been confirmed in humans. Studies often lack infant plasma concentrations, which could explain associations with growth, and even with infant plasma available, concentrations might reflect endogenous produced hormones rather than orally ingested. Finally, the hormones might correlate with other nutrients in HM, which may be the true driver of associations seen.

### Materials and methods

Colostrum samples were collected between 24 and 72 h after birth, which was chosen for flexibility allowing most mothers to collect the sample. The variation in HM concentration of each hormone was largest for colostrum compared to any of the other visit’s samples, indicating either a physiologically greater variation in colostrum compared to mature milk, or that some samples might have been transition milk with lower concentrations compared to colostrum.

The use of mediation analyses holds the assumptions that no unmeasured confounding should influence (a) the exposure-outcome association, (b) the mediator-outcome association and/or (c) the exposure-mediator association. Counterfactual-based mediation analyses further assumes that (d) no unmeasured mediator-outcome effect are affected by the exposure, which is necessary in defining direct and indirect effects (33). In HM research, and especially observational studies using biomarkers in blood and/or HM, the assumptions are a concern as confounding can rarely be completely removed. Contrary to randomized controlled trials, the exposure in the present study (BMI/FMI groups) was not randomized, which increases the risk of unmeasured confounding. However, infant age and mean-centered age were assumed to be confounders in step a-c above and appropriately adjusted for. Furthermore, the risk of exposure-induced mediator-outcome confounding (d) is markedly reduced, when the mediator appears shortly after the exposure (58), which is the case in the present study.

### Strengths and limitations

The main strength of the present study is the longitudinal design combined with the comprehensive data systematically collected from both mother and infant. Compared to similar studies, sample size is comparable, especially when using multilevel modelling which is robust to missing data points.

A main limitation in the present study is the use of assays yet to be validated for hormone analyses in human milk. Our results of HM leptin are in the low range of the wide range of concentrations presented in the review by Suwaydi *et al.*, whereas insulin concentrations are within the range and adiponectin is lower than the presented range (59). For plasma concentrations of the ARHs, our results of leptin and insulin are comparable to the literature, while adiponectin is lower than reported elsewhere (60). However, the ratio between plasma and HM adiponectin is similar to previously reported (59–61), indicating that it might be the standard of the assay rather than the matrix that causes the low concentrations. As our results show a systematic underestimation compared to current literature, the underestimation is less likely to affect our results of associations between maternal adiposity and HM ARH as well as the mediated proportions found in mediation analyses. Suwaydi and colleagues further mention that factors such as time of collection (e.g., morning or afternoon), sample type (e.g., colostrum or mature milk), sampling (pre- and post-feed or complete breast expression) influence the concentrations measured and challenge comparison between studies. These considerations supplement the need for validated assays for analysis of ARH in HM.

Furthermore, it is a limitation that we generally had a reduced sample size in the group of mothers with overweight, and for infants with blood samples, which likely reduced power to detect significant associations. Therefore, studies are highly encouraged to include both maternal and infant blood samples as well as milk samples in order to more comprehensively investigate the signaling mechanisms through human milk. Using BMI for classification of overweight may overestimate adiposity in certain populations (62), which could be relevant in the present study as we find more women categorized as overweight compared to having excessive fat mass estimated by BIA. This is a limitation, and our results show discrepancy in estimates of these two exposures. This could reflect true differences in group sizes or potentially an overestimation of adiposity when using BMI. The discrepancy could hypothetically be explained by an underestimation of FM caused by overestimation of FFM due to breastmilk and hydration status effecting bioelectrical impedance assessment. However, fat mass has been shown to be overestimated in lactating women using the BIA method compared to the golden standard of deuterium oxide method (63), which emphasizes the relevance of studies determining body composition in lactating women using more precise techniques.

In conclusion, our results illuminate the influence of maternal adiposity on HM ARH composition possibly through obesity related low-grade inflammation and further describe the influence on infant outcomes. The present study is the first to use counterfactual-based mediation analyses, and thereby provides a novel statistical approach in the investigation of associations between maternal predictors of ARH in HM and infant growth. Future studies are needed to confirm the findings in large observational studies and by using same methodological approaches.

## Data availability statement

The datasets for this article are not publicly available due to concerns regarding participant/patient anonymity. Requests to access the datasets should be directed to the corresponding author.

## Ethics statement

This study involved human subjects including under-aged subjects (<18 years old). Written informed consent was obtained from all subjects, and all custody holders of the under-aged subjects gave written informed consent for participation. The study was approved by the National Committee on Health Research Ethics on 1st August 2017 (Journal number: H-17015174).

## Author contributions

LA, KM, SC, AL, and CM participated in designing and conducting the study. HF analyzed the biological samples. SC and JL analyzed the data statistically. SC wrote the manuscript and had primary responsibility for the final content. All authors have read and approved the final manuscript.

## Abbreviations

ARH: appetite-regulating hormones
BMI: body mass index
CV: coefficient of variance
CI: confidence interval
CRP: C-reactive protein
DM: diabetes mellitus
EBF: exclusive breastfeeding/exclusively breastfed
FFM: fat-free mass
FFMI: fat-free mass index
FM: fat mass
FMI: fat mass index
HM: human milk
IQR: Interquartile range
MILQ: “Mothers Infants and Lactation Quality” study
MP: mediated proportion
NA: non-detectable
RQ: research question
SD: standard deviation
TNF-α: tumor-necrosis factor-α
V2, V3, V4: Visit 2, Visit 3, Visit 4
WHO: World Health Organization
WLZ: Weight-for-length Z-scores.
