# Supplementary material for: Associations between maternal adiposity and appetite-regulating hormones in human milk are mediated through maternal circulating concentrations and might affect infant outcomes
Source: Front Nutr. 2022 Nov 4;9:1025439. doi: 10.3389/fnut.2022.1025439 (PMC9673480; doi:10.3389/fnut.2022.1025439)
Supplement: Supplementary file 1 [file Table_1.pdf]

SUPPLEMENTARY TABLES

Table S1. Concentrations of leptin, insulin and adiponectin in human milk and in maternal and infant plasma.

Human milk	**V1 (*n*=146)**	**V2 (*n*=206)**	**V3 (*n*=197)**	**V4 (*n*=200)**
**Leptin (pg/mL)**				
Mean (SD)	757 (836)	272 (294)	262 (439)	188 (264)
Median (IQR)	482 [237, 909]	161 [75, 374]	108 [46, 285]	118 [60, 249]
NA's (%)	16 (10.9)	60 (29.1)	65 (33.0)	88 (44.0)
**Insulin (pg/mL)**				
Mean (SD)	576 (548)	219 (93)	238 (101)	238 (102)
Median (IQR)	422 [272, 665]	203 [153, 267]	219 [164, 286]	212 [170, 296]
NA's (%)	6 (4.1)	0 (0.0)	3 (1.5)	11 (5.5)
**Adiponectin (ng/mL)**				
Mean (SD)	39.0 (83.1)	2.8 (1.8)	2.3 (1.7)	2.7 (2.4)
Median (IQR)	8.3 [4.7, 25.8]	2.4 [1.6, 3.7]	1.8 [1.2, 2.7]	2.0 [1.3, 3.1]
NA's (%)	10 (6.8)	2 (1.0)	7 (3.6)	13 (6.5)

Maternal plasma	**V2 (*n*=223)**	**V3 (*n*=209)**	**V4 (*n*=201)**
**Leptin (ng/mL)**			
Mean (SD)	10.2 (14.0)	7.8 (9.4)	8.8 (10.2)
Median (IQR)	4.8 [2.1, 12.4]	4.4 [1.8, 10.4]	5.1 [2.5, 11.2]
NA's (%)	1 (0.4)	3 (1.4)	0 (0.0)
**Insulin (pg/mL)**			
Mean (SD)	156 (90)	190 (107)	161 (102)
Median (IQR)	131 [93, 180]	163 [109, 254]	127 [88, 201]

NA's (%)	1 (0.4)	0 (0.0)	0 (0.0)
**Adiponectin (ng/mL)**			
Mean (SD)	4.6 (2.1)	7.3 (4.1)	7.0 (8.3)
Median (IQR)	4.4 [3.1, 5.7]	6.9 [5.0, 9.2]	4.0 [2.7, 6.7]
NA's (%)	2 (0.9)	3 (1.4)	1 (0.5)

Infant plasma	**V2 (*n*=105)**	**V3 (*n*=47)**	**V4 (*n*=52)**
**Leptin (ng/mL)**			
Mean (SD)	2.7 (2.8)	3.0 (4.1)	3.1 (2.7)
Median (IQR)	1.4 [0.7, 3.3]	1.6 [1.0, 3.2]	2.7 [1.1, 4.7]
NA's (%)	72 (68.6)	9 (19.1)	22 (42.3)
**Insulin (pg/mL)**			
Mean (SD)	153 (82)	91 (45)	120 (86)
Median (IQR)	139 [94, 180]	78 [61, 111]	85 [71, 152]
NA's (%)	2 (1.9)	9 (19.9)	22 (42.3)
**Adiponectin (ng/mL)**			
Mean (SD)	24.7 (9.5)	19.3 (5.6)	11.7 (4.7)
Median (IQR)	23.4 [17.8, 28.8]	19.6 [15.5, 22.9]	10.1 [9.0, 13.8]
NA's (%)	20 (19.0)	9 (19.1)	21 (40.4)

Results are given both as mean (SD) and as median [IQR] Non-detectable data are given as counts (%). Visit 2 (V2) = 1-3.49 months, V3 = 3.5-6.0 months, V4 = 6-8.49 months postpartum. IQR=Interquartile range, NA=Not detectable, SD=standard deviation.

Table S2: Definitions of total, direct and indirect effects in mediation analyses using BMI (exposure) as a categorical variable, i.e. mothers with normal-weight (BMI<25 kg/m^2) or with overweight (BMI≥25kg/m^2). The same definitions applies to the analyses using FMI (exposure) as a categorical variable, i.e. mothers with normal fat mass (FMI≤9) or excessive fat mass (FMI>9).

1. c_1'(normal-weight) = Direct effect for mothers with normal-weight = how much HM leptin would differ comparing the two hypothetical situations in which all mothers had normal-weight or all mothers had overweight, but maternal plasma leptin were kept at a level it would have been for mothers with normal-weight

2. a_1b_1(normal-weight) = Indirect effect for mothers with normal-weight = how much HM leptin would differ if all mothers had overweight, but maternal plasma leptin was shifted to a level it would have if they all had normal-weight

3. c_1'(overweight) = Direct effect for mothers with overweight = how much HM leptin would differ comparing the two hypothetical situations in which all mothers had normal-weight or all mothers had overweight, but maternal plasma leptin were kept at a level it would have been for mothers with overweight

4. a_1b_1(overweight) = Indirect effect for mothers with overweight = how much HM leptin would differ if all mothers had normal-weight, but maternal plasma leptin was shifted to a level it would have if they had overweight

5. c_1 = Total effect (TE) = the effect of exposure on outcome = how much HM leptin would differ comparing the two hypothetical situations in which all mothers were normal-weight or all mothers were overweight.

$$TE = c_1'(\text{normal-weight}) + a_1b_1(\text{overweight}) = c_1'(\text{overweight}) + a_1b_1(\text{normal-weight})$$

Table S3. Mediation analyses of the association between either **a)** maternal body mass index or **b)** maternal fat mass index, and human milk concentrations of leptin and insulin, respectively, as outcome variables with maternal plasma concentrations of the respective hormones as mediating variables in each model (Research Question 1).

	**Log-HM leptin[1] (n=221)**			**Log-HM insulin[2] (n=221)**		
**PATH (RQ1)**	ESTIMATE	95% CI	MP	ESTIMATE	95% CI	MP
a) Maternal BMI						
a_1b_1 (indirect effect); *BMI<25kg/m²*	0.48***	[0.30-0.67]	0.29***	0.0039	[-0.00017-0.01]	0.15
a_1b_1 (indirect effect): *BMI≥25kg/m²*	0.81***	[0.45-1.21]	0.51***	-	-	-
c_1' (direct effect): *BMI<25kg/m²*	0.79**	[0.23-1.32]		0.021**	[0.0075-0.04]	
c_1' (direct effect): *BMI≥25kg/m²*	1.12***	[0.73-1.50]		-	-	
c_1 (total effect)[#]:	1.60***	[1.18-2.13]		0.025**	[0.010-0.04]	
b) Maternal fat-mass index						
a_1b_1 (indirect effect); *FMI≤9*	0.41***	[0.22-0.62]	0.26***	0.010***	[0.0034-0.020]	0.32*

a_1b_1 (indirect effect): *FMI>9*	0.24	[-0.097-0.56]	0.14	-	-	-
c_1' (direct effect): *FMI≤9*	1.30***	[0.58-2.03]		0.023*	[0.0017-0.040]	
c_1' (direct effect): *FMI>9*	1.13***	[0.63-1.74]		-	-	
c_1 (total effect):	1.54***	[1.04-2.05]		0.033*	[0.010-0.060]	

All models included in the mediation analyses were adjusted for infant sex, visit and mean-centered age. Estimates for a_1b_1 describes the mediated effect (indirect effect) of either maternal BMI or FMI (exposure variables) on HM leptin and insulin (outcome variables), respectively, through maternal plasma concentrations of the respective hormone in each model. Estimates for c_1 describes the total effect of exposure on outcome (direct + indirect effect) and estimates for c_1' is the direct effect of exposure on outcome, when removing the mediated effect through maternal plasma concentrations. Mediation analyses are made only for significant total effects in the initial model [1]A significant exposure-mediator interaction in the mediation analyses of the association between maternal BMI and FMI, respectively, and HM leptin resulted in separate estimates for the indirect and direct effect depending on maternal BMI/FMI groups (i.e. BMI <25 or ≥25 kg/m^2 and FMI ≤9 or >9). [2]No exposure-mediator interaction was present in the association between maternal BMI and HM insulin, and estimates of indirect and direct effects are shown using maternal BMI or FMI as continuous variables.

*BMI=Body mass index, CI=confidence interval, FMI=Fat mass index, HM=Human milk, MP=Mediated proportion, RQ=Research Question; p<0.001:'***',p<0.01:'**', p<0.05: '*'.*
